# Poisoning Cases Reported to Poison Information Centre, Ahmedabad, India: A Three Year Observational Study

**DOI:** 10.5195/cajgh.2020.471

**Published:** 2020-03-31

**Authors:** Avinash Pagdhune, Kundan Kunal, Kanubhai Amrutlal Patel, Aswin Bhailalbhai Patel, SukhDev Mishra, Rajendra Palkhade, Jaseer Muhamed

**Affiliations:** 1 Poison Information Centre, ICMR-National Institute of Occupational Health, Ahmedabad, Gujarat, India; 2 Biostatistics and Data Management, ICMR-National Institute of Occupational Health, Ahmedabad, Gujarat, India; 3 Animal Facility, ICMR-National Institute of Occupational Health, Ahmedabad, Gujarat, India; 4 Biochemistry, ICMR Regional Occupational Health Centre (Southern), Karnataka, India

**Keywords:** Trend of poisoning, Pesticide poisoning, Organophosphorous, Suicide

## Abstract

**Introduction:**

Morbidity and mortality associated with pesticide poisoning is a major public health issue, especially in lower and middle income countries, including India. Timely understanding of poisoning trends is required for improved prevention. The objective of the present study was to analyze the trend of poisoning cases in Ahmedabad, India in the period of 2015–2017.

**Methods:**

Detailed history, including demographic data, risk factors, poisoning history, agents involved, and occupational influence were collected for poisoning cases reported to the Poison Information Centre in Ahmedabad. Cholinesterase activity and HPTLC method for detection of sanguinarine in urine were used to investigate the agents of poisoning. Non-parametric tests, such as Chi-square test and Mann-Whitney U Test were applied to test statistical significance between the groups. All statistical analysis was carried out using IBM SPSS Statistics for Windows, Version 26.0. Armonk, NY: IBM Corp.

**Results:**

A total 1373 poisoning cases were investigated. The incidence and fatality rate was found to be higher in males compared to females (M/F ratio 1.89:1). About 91.62% of the poisoning were through the oral route. Erythrocyte cholinesterase activity assay results indicated that 41.29% of the cases were due to organophosphorus/carbamate poisoning. Insecticides were found to be the agent of poisoning in 26.29% cases, and 11.07% of all the cases were agricultural workers. Poisoning with medications, household pesticides and chemicals were also reported. Few cases of food poisoning with sanguinarine were detected.

**Conclusions:**

The data presented here suggest that pesticides used for agriculture are the major source of poisonings. Implementation of usage guidelines, educating farmers and vulnerable population, and finding novel alternatives for highly toxic chemicals may be helpful in decreasing the number of poisoning cases.

Morbidity and mortality due to pesticide poisoning is a major public health issue, especially in lower and middle income countries.^1^ According to the National Crime Records Bureau of India, the official estimate of suicides resulted from insecticide poisoning was 10.4% of the total suicide cases in 2014.^2^ This estimate is more likely to be lower than the actual number because of under-reporting of suicide cases and exclusion of poisoning cases associated with rodenticides, fungicides and herbicides.^1^ An estimate of 11.3% of total suicide cases due to insecticide poisoning was also reported in lower and middle income countries of the South-East Asia region.^1^ In 2010, there was reports of incidence of 38.8% of suicide by pesticide ingestion in India.^3^ Among the Indian states, Gujarat state accounts for 5.4% of the suicide cases, and this is above the national average of the country.^4^

To date, considerable efforts have been taken by the World Health Organization (WHO) and different countries to reduce the incidence of poisoning cases. One such initiative is an IPCS INTOX program by WHO in 1988, which promoted chemical safety by establishing poison information centers, which is a global endeavor to promote chemical safety by the introduction and support of poison information centers. The program aimed to harmonize the collection of poisoning data, training and sharing of information related to poisoning within member countries.^5^ In accordance with this program, India currently has six poison information centers. The Central Insecticides Board & Registration Committee, under the Department of Agriculture and Co-operation, Government of India, is engaged with registration of pesticides used in agriculture in the country. Its activities involve banning the pesticides and chemicals that are hazardous to health and the environment, establishing guidelines for registration of new pesticides, establishing minimum infrastructure requirements for pesticide manufacturing, and setting guidelines for the export and import of pesticides.^6^ Still, morbidity and mortality from poisoning continue to be a major public health concern in the country.

The incidence of poisoning depends on several factors including socioeconomic status, culture and religion, educational status, agricultural practices and knowledge of pesticides and other poisonous substances, extent of industrialization, and geographical conditions.^7^ Consequently, the epidemiology of poisoning cases may vary depending on geographic location, while understanding of the pattern and trend of poisoning in a particular area is necessary for efficient design and implementation of sustainable prevention and control strategies. Previous study conducted in Delhi, India, identified that household chemicals followed by drugs, agricultural pesticides, and industrial chemicals were the major agents of poisoning.^8^ The objective of this study was to understand the trend of the poisoning cases arising from human exposure to different poisons, including pesticides, during 2015–2017 to inform preventive measures that may help to reduce future incidence of poisoning cases. The article describes the trend of poisoning cases reported to a poison information center at Ahmedabad, Gujarat between 2015 and 2017.

## Methods

### Fatality rate due to poisoning

In order to understand the annual mortality trend due to poisoning, the rate of fatality due to poisoning was obtained. It was calculated from the number of fatalities due to poisoning and the total number of poisoning cases reported.

### Collection of epidemiological data

A detailed history of poisoning cases was taken for each of the poisoning cases reported to the poison information center from January 1, 2015 to December 31, 2017. The ethical committee approval was obtained from institutional ethics committee at the National Institute of Occupational Health, Ahmedabad, Gujarat, India. Informed oral consent was obtained from each patient and/or their guardians for the use of their data in this research. The proforma for patient history included patients’ personal data such as age, sex, marital status, education and geographical area of residence. Occupation of the patient was noted to identify any occupational exposures. The poison severity score (none, minor, moderate or severe) as per Persson et al., 1998^9^ was documented at the time of admission to the hospital and was obtained for this study. Efforts were taken to document information on the chemical involved in the poison. Other information like chronicity of poisoning, route of poisoning, such as oral, inhalation, dermal exposure, etc. were obtained from the attending physician. Information such as the consciousness of the patient at the time of admission, as well as requirement of ventilator support, were also documented for each patient.

### Estimation of cholinesterase activity

Cholinesterase activity was used as the diagnostic tool for acute organophosphorus poisoning. Plasma and/or RBC cholinesterase activity was measured using modified *Ellman's* spectrophotometric method.^10^ Briefly, plasma was added and mixed with 5,5’-dithiobis-(2-nitrobenzoic acid) reagent (Sigma) and acetyl thiocholine substrate (Sigma) was added to the mixture. The yellow color developed was measured at 410nm using a spectrophotometer (Cary 100 Bio, Varian), and cholinesterase activity was expressed in units per liter of plasma. The value of cholinesterase activity was then compared with biological reference range generated in-house.

### Detection of sanguinarine

Sanguinarine in urine samples of poisoning cases were detected by HPTLC method according to methodology published by Shenolikar et al.^11^ Briefly, sanguinarine in urine samples was extracted with 1% acetic acid in chloroform and spotted onto TLC plates (Sigma) along with standard and developed with butanolacetic acid-water in the ratio 63:10:27 by volume. The plates were then observed under ultraviolet light and the golden-yellow fluorescent band in line with standard sanguinarine spot was identified and evaluated as sanguinarine in the sample.

### Statistical analysis

Non-parametric tests such as Chi-square test and Mann-Whitney U Test were applied to test statistical significance between the groups. All statistical tests were carried out at 5% level of significance. All statistical analyses were carried out using IBM SPSS Statistics for Windows, Version 26.0. Armonk, NY: IBM Corp.

## Results

**Figure 1.**
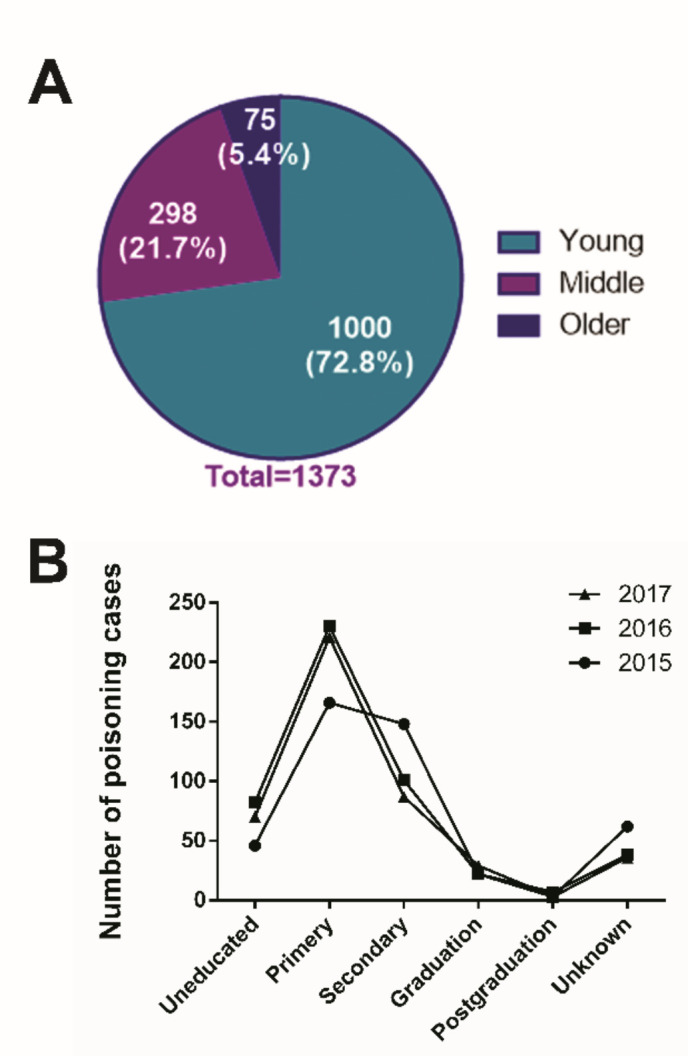
The pie chart shows the number of poisoning incidences in young, middle and older aged subjects (A) and educational status of the patients (B) between 2015 and 2017.

A total of 1373 poisoning cases was reported to the poison information center, with the highest number in the year 2016 (480 cases). Although the fatality rate due to poisoning in Ahmedabad showed an increasing trend annually (Table 1), it was not statistically significant. The male to female ratio was 1.4, 1.8 and 2.7 in the years 2015, 2016 and 2017, respectively, indicating the increasing trend of incidence of poisoning in males. Also, the fatality rate was significantly high in males compared to females. The distribution of poisoning cases with respect to the age of patients^12^ represented as young (age ≤35 years), middle age (36–55 years) and older (age ≥56) are shown in Figure 1A.

**Table 1.** Data on poisoning cases reported to poison information center

	2015	2016	2017	Total
**Number of cases**	447	480	446	1373
** Males**	262	310	326	898
** Females**	185	169	120	474
** Third gender**	0	1	0	1
**Male to female ratio**	1.42	1.83	2.72	1.89
**Number of fatalities**	27	42	43	112
** Number of fatalities (male)**	19	30	36	85^*^
** Number of fatalities (female)**	8	12	6	26
**Percentage of fatal rate**	6.04%	8.75%	9.64%	8.15

* significant at p-value 0.10

The highest level of incidence was observed in the younger age group. Figure 1B shows the education status of the patients. The educational status of 136 patients was not known. The trend was similar in all the three years of the study.

**Figure 2.**
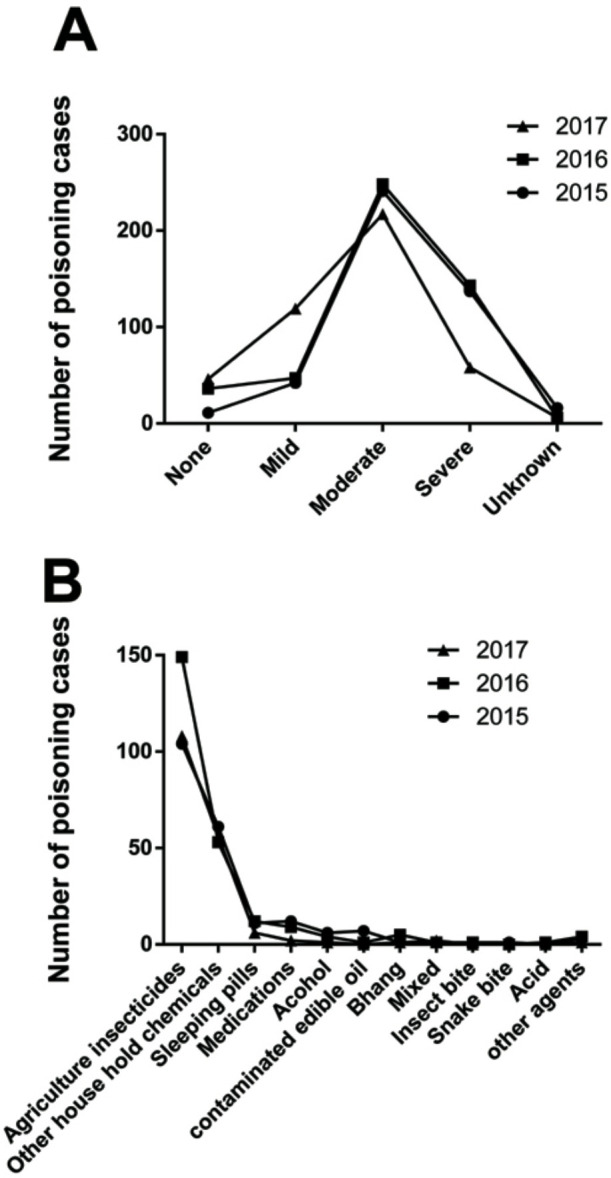
The annual trend of poisoning cases in relation to the severity of cases at the time of admission to hospital (A) and the agents involved in poisoning (B) between 2015 and 2017.

The poison severity score at the time of admission to the hospitals revealed that 338 cases were severe (24.61%), while 51.42% cases were of moderate severity. Figure 2A shows the three-year trend of severity at admission, indicating similar trends in these three years. During the course of treatment, 289 (21.04%) poisoning cases required ventilator support. A total of 47 patients suffered from limb paralysis due to poisoning during this time period. The route of exposure was oral in the majority of the cases with 89.5%, 91.6% and 93.7% cases reported in the years 2015, 2016 and 2017, respectively. A total of 23 poisoning cases were reported due to poisoning through inhalation of toxic agent. Four cases of poisoning through dermal exposure were also documented. Figure 2B illustrates the trend of various chemical agents used for poisoning. The trend was similar in all three years, except the occurrence of seven edema cases due to contaminated edible oil in the year 2015, followed by one case in 2016. There were no edema cases reported in the year 2017. The chemical nature of the poison was not known to the investigators in 52.5% of the cases. Agricultural insecticides were found in 26.29% cases, followed by household chemicals like phenyl, rodenticides, mosquito repellents and bleaching powder, together comprising about 12.31% of the poisoning cases.

**Figure 3.**
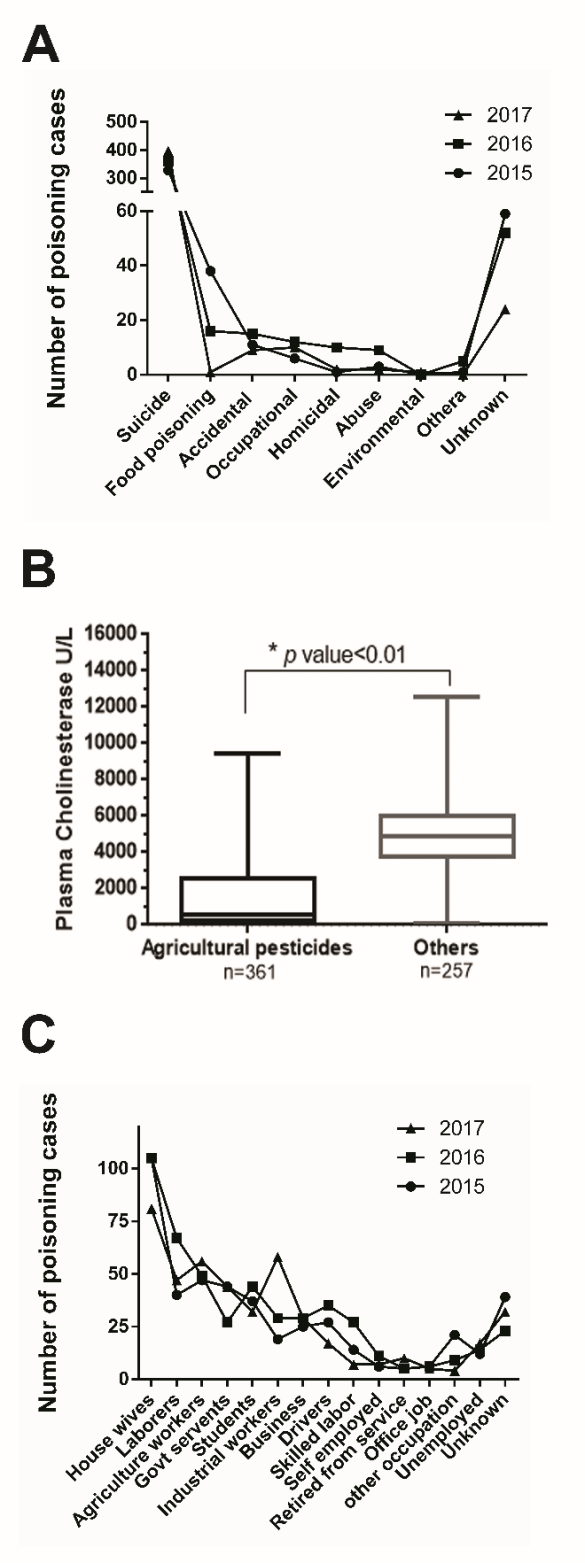
The trend of poisoning 2015–2017 in relation to the circumstances of poisoning (A), the box plot showing plasma cholinesterase activity in victims of poisoning due to agricultural pesticides in comparison to other known causes (B), and the occupation of patients (C).

The trend in relation to circumstances of poisoning cases reported is shown in Figure 3A. Most of the cases were suicidal in nature (73.4%, 75.2% and 88.8% in the years 2015, 2016 and 2017, respectively) with intentional oral intake of poison at home. Agricultural chemicals stored in home were found to be the poisoning agent in 28.18% of the suicide cases.

Chemical agents included acephate, phorate, adrin, carbofuran, celphos, chlorophos, chorpyriphos, cypermethrin, DDT, gamexin, dimethoate, imidochloprid, malathion and monocrotophos. The list also includes some insecticides with local trade names whose chemical composition was not clear. Analysis of cholinesterase activity in these patients revealed 571 (52.57%) cases with reduced plasma cholinesterase activity and 428 (39.41%) cases with reduced RBC cholinesterase activity, indicating the widespread use of organophosphorus chemicals for intentional poisoning. Plasma/serum cholinesterase activity and RBC cholinesterase activity reduced in 49.67% and 41.29% of all the cases. The cholinesterase values of poisoning cases due to agricultural pesticides were significantly lower than that of poisoning due to causes other than agricultural pesticides (p value <0.05) as shown in Figure 3B.

Thirteen cases were homicidal in nature (1, 10 and 2 cases in the year 2015, 2016 and 2017, respectively). Six of them had reduced RBC cholinesterase activity with normal level of plasma cholinesterase activity, and another 2 cases had very low level of plasma cholinesterase activity.

About 55 cases of food poisoning and 35 cases of accidental poisoning were recorded in the study period. The number of food poisoning cases showed a decreasing trend with 38, 16 and 1 cases in the year 2015, 2016 and 2017, respectively. Paralysis of both limbs of the patients were present in 18 food poisoning cases reported in 2015. Among these, eight were suspected cases of argemonium oil consumption, and HPTLC analysis of urine sample of the patients revealed the presence of sanguinarine in three cases. About 38.18% of the food poisoning cases showed reduction in the plasma cholinesterase activity, and 65.45% showed reduced RBC cholinesterase activity.

Fourteen cases were associated with the abuse of toxic substances. Four of them were with Bhang, a locally available cannabinoid substance, and one case with locally made alcohol. Three of these patients showed reduced plasma/RBC cholinesterase activity indicating the possibility of organophosphorus poisoning.

Twenty-eight cases were reported with poisoning related to occupational exposures. All of these were acute poisoning cases, out of which 16 cases were from inhalation of toxicant, and 11 cases were oral poisoning cases. At least 21 of them had reduced plasma cholinesterase activity, and 15 cases had reduced RBC cholinesterase activity, indicative of organophosphorus poisoning in occupational settings. Figure 3C shows the trend of poisoning with respect to the occupation of patients. In all three years, housemakers constituted the highest number of poisoning cases (21.2%), followed by laborers (11.2%), agricultural workers (11.0%) and industrial workers (7.7%). The number of industrial workers exposed to poison at workplace was 19, 29 and 56 in the years 2015, 2016 and 2017, respectively.

## Discussion

This study presents the trend of poisoning cases reported to poison information center, Ahmedabad from 2015 through 2017. The fatality rate due to poisoning was found to be similar from 2015 to 2017, underlining the importance of poisoning as a public health concern. This rate may be an underestimate because of under-reporting of deaths due to poisoning. The incidence and fatality due to poisoning was higher in males. A similar study conducted in a tertiary hospital in Karnataka state, India also reported higher incidence (75.4%) of poisoning among males compared to females.^13^ However, there are studies that reported higher incidence in females^14^, as well as comparable incidence in males and females.^15^ This might be attributed to the difference in the cultural, lifestyle, occupational and socioeconomic nature of the population studied.

In the present study, 11.07% of the population comprised of agricultural workers, and in 26.29% of the reported cases, the poisoning was associated with insecticides used in the agriculture sector. This is a matter of concern, especially in the context of recent reports of fatal occupational poisoning of 45 agricultural workers in the Bt-cotton plantations of Maharashtra state.^16^ A similar method of cotton cultivation is being practiced in Gujarat too.^17^ Though Bt-cotton plants are supposed to be resistant to insect cotton ballworms, new reports are suggestive of development of resistance in ballworms.^18^ This leads to heavy insecticide use in Bt-cotton plantations and results in occupational poisoning in agricultural workers, as well as higher incidence of suicide. A notable study among 127 acute poisoning cases in Maharashtra reported 48.8% cases in agriculture workers.^19^ The cause of higher incidence of poisoning cases among farmers of Vidarbha region of Maharashtra was identified as a complex interplay of social, political and environmental factors. Relief packages, as well as implementation of mental health programs at the regional level to offer support and counselling to vulnerable population, may prevent the incidence of poisoning cases in future.^20^ Similarly, the use of personal protective equipment and safety guidelines in the use of pesticides may also aid in reducing the occupational poisoning, especially in workers who spray the insecticide solution. Proper training in integrated pest management has been proven as an effective strategy in reducing the number of poisoning cases in farm workers of South India.^21^

Organophosphorus pesticides are the most common cause of poisoning in agricultural workers and other unskilled workers.^22^ Currently, cholinesterase activity in serum/plasma/RBC is used as most reliable tests for organophosphorus poisoning.^23^ This study revealed reduced cholinesterase activity in plasma of victims exposed to agricultural pesticides compared to cases due to other means of poisoning, emphasizing the prevalence of poisoning with pesticides such as organophosphorus chemicals/carbamates in the community. This observation warrants the need for replacing highly poisonous organophosphorus chemicals with less toxic chemicals. We would also like to emphasize the need to regulate the supply of toxic chemicals, implementation of usage guidelines, and banning of highly toxic pesticides.

Poisoning with alcohol is relatively low in Gujarat.^24^ In this study, we came across 11 alcohol poisoning cases, which comprised only 0.80% of total poisoning cases reported. This could be the result of Bombay prohibition bill passed in 1949 and subsequent amendments by the state government, according to which liquor is prohibited by law in Gujarat state.^25^ However, previous studies on the drunkenness at Ahmedabad civil hospital have reported some episodes of alcohol poisoning, and the alcohol prohibition by law did not necessarily change the behavior of people towards the usage of alcohol.^24^

Eight cases of poisoning through contaminated edible oil was reported. Out of this, the presence of sanguinarine, a toxic alkaloid present in *Argemone mexicana* seeds was detected in the urine of three patients, suggestive of edema (epidemic dropsy), in the year 2015. Edema usually occurs in the form of an epidemic affecting a population that consume edible oil adulterated with *Argemone mexicana* oil.^26^ In the year 2012, thirteen cases of edema were reported from Panchmahal district of Gujarat.^27^ Hence, even though a small incidence of the disease was reported to the poison information center in the year 2015, the data points towards the need of active toxicovigilance and anti-food adulteration activities. Moreover, the toxicology laboratories have to be strengthened for timely detection and diagnosis of edema in future.

Household chemicals like insecticides, rodenticides, phenyl, bleaching powder and mosquito repellents constituted 12.31% of the poisoning cases. Previous studies also reported a higher incidence (44%) of poisoning due to such household chemicals.^8^ This difference in the trend of poisoning might be attributed to the differences in the culture, education status and availability of other toxicants for suicide purpose. The probability of poisoning is inversely proportional to the education level.^28^ The education status of patients in this study also showed that the poisoning incidence is high among the uneducated or less educated population (Figure 1B). Therefore, increasing the awareness on poisonous substances may be effective in reducing the number of poisoning cases in future.

According to WHO, pesticide poisoning accounts for the most of the global suicides, and the majority of them occur in lower and middle income countries.^29^ The present study also shows that agriculture pesticides are the major means of poisoning and related fatality. A multifaceted approach with legislation to ban highly toxic pesticides, improvements in medical management of poisoning cases, awareness and storage guidelines may help to reduce the incidence of poisoning, as found effective in Srilanka.^30^ More effective toxicovigilance by the regulatory agencies also can contribute to bring down the poisoning cases to minimum level.^31^

The limitation of this study is that it is possible that not all poisoning cases have been reported to the center. The availability and nature of particular antidotes were also not documented in this study. These are very important for assessment of health resources required to deal with poisoning cases in a particular region. Therefore, strengthening the reporting procedures of poisoning cases to the poison information center and special attention to availability of antidotes is an important aspect of future work in this important area.

Poisonings with toxic chemicals continue to be a major health concern in Gujarat, and poison information centers play a crucial role in reducing the rate of poisoning. The data presented in this paper suggest that pesticides used in agriculture were a major source of poisoning between 2015 and 2017. Highly toxic chemicals used in agriculture should be either banned or given to farmers with strict usage guidelines and documentation explaining the risks involved. Most of the poisoning cases reported here were suicidal in nature, indicating the need for effective measures to prevent the suicidal tendency and improve psychological health in the community. The epidemiological trend of poisoning cases presented in this paper may be helpful in reducing the incidence of poisoning cases in future.
